# Maintained Properties of Aged Dental Pulp Stem Cells for Superior Periodontal Tissue Regeneration

**DOI:** 10.14336/AD.2018.0729

**Published:** 2019-08-01

**Authors:** Linsha Ma, Jingchao Hu, Yu Cao, Yilin Xie, Hua Wang, Zhipeng Fan, Chunmei Zhang, Jinsong Wang, Chu-Tse Wu, Songlin Wang

**Affiliations:** ^1^Molecular Laboratory for Gene Therapy & Tooth Regeneration, Beijing Key Laboratory of Tooth Regeneration and Function Reconstruction, Capital Medical University, School of Stomatology, Beijing, China; ^2^Department of Experimental Hematology, Beijing Institute of Radiation Medicine, Beijing, China; ^3^Department of Biochemistry and Molecular Biology, Capital Medical University School of Basic Medical Sciences, Beijing, China

**Keywords:** mesenchymal stem cells, senescence, inflammation, periodontitis

## Abstract

Owing to excellent therapeutic potential, mesenchymal stem cells (MSCs) are gaining increasing popularity with researchers worldwide for applications in tissue engineering, and in treatment of inflammation-related and age-related disorders. However, the senescence of MSCs over passaging has limited their clinical application owing to adverse effect on physiological function maintenance of tissues as well as disease treatment. An inflammatory microenvironment is one of the key contributors to MSC senescence, resulting in low regeneration efficiency. Therefore, MSCs with high resistance to cellular senescence would be a benefit for tissue regeneration. Toward this end, we analyzed the senescence properties of different types of stem cells during culture and under inflammation, including dental pulp stem cells (DPSCs), periodontal ligament stem cells (PDLSCs), bone marrow mesenchymal stem cells (BMMSCs), and adipose-derived stem cells (ADSCs). Overall, the DPSCs had higher proliferation rates, lower cellular senescence, and enhanced osteogenesis maintenance compared to those of non-dental MSCs cultured from passage three to six. The expression profiles of genes related to apoptosis, cell cycle, and cellular protein metabolic process (contributing to the cell self-renewal ability and metabolic processes) significantly differed between DPSCs and BMMSCs at passage three. Moreover, DPSCs were superior to BMMSCs with regards to resistance to lipopolysaccharide-induced apoptosis and senescence, with enhanced osteogenesis *in vitro*, and showed improved periodontal regeneration after injection in a miniature pig periodontitis model *in vivo*. Overall, the present study indicates that DPSCs show superior resistance to subculture and inflammation-induced senescence and would be suitable stem cells for tissue engineering with inflammation.

Mesenchymal stem cells (MSCs), possessing self-renewal and multi-lineage differentiation potential as well as immunomodulation ability, have attracted the interest of researchers and clinicians. Owing to their versatile properties, there have been several attempts and explorations for MSCs-based therapy, including for tissue regeneration or injury repair, and in the treatment of inflammation and autoimmune diseases [1] or aging disorders [2]. However, the therapeutic potential and efficiency depend on the interaction between MSCs and their microenvironment or niche [3]. A healthy microenvironment will sustain the bio-function of MSCs, whereas senescence will readily occur under a pathological condition such as inflammation. Therefore, studying the relationship between stem cells and health can uncover the impact of aging on the stem cell functionality and indicate the importance of stem cell capacity to health maintenance [4]. In addition, stem cell aging (also described as senescence) with proposed biological features such as shortening of telomeres, DNA damage, and epigenetic alterations of transcriptional regulation [5], results in irreversible cell proliferation arrest [6] and depletion, as well as failed differentiation, which obviously impairs the potential of MSCs for disease therapy [7]. Earlier-passage MSCs have been demonstrated to have better colony efficiency compared to later-passage MSCs [8]. The classic features characterizing the senescence phenotype of MSCs include growth arrest in the G1 phase of the cell cycle, an enlarged or flattened morphology, increased expression of senescence-associated β-galactosidase (SA-β-gal) and senescence-associated lysosomal α-L-fucosidase (SA-α-Fuc), and decreases in the levels of some surface markers such as Stro-1 or CD106 [9]. Importantly, the senescence of MSCs can be induced by both the culture and regeneration microenvironment, which would impair the effectiveness of stem cell-based regenerative therapy [10]. Periodontitis is one of the most prevalent chronic infectious diseases of the soft and hard tissues supporting the teeth [11], affecting 15-50% of adults worldwide [12]. Periodontitis is the leading cause of tooth loss in adults, with a significant impact on oral health and overall quality of life. Indeed, the traditional treatments of periodontitis, including debridement of the root surface to induce healing, guided tissue regeneration, and bone graft placement, are associated with a relatively high degree of variability in clinical outcome [13], and the curative effect remains unsatisfactory. Owing to their potential for differentiation and sensitivity to local paracrine activity, MSCs may exert multiple effects against periodontitis, including neovascularization, immunomodulation, and tissue regeneration [14]. Recent advances in regenerative medicine have shown that MSCs-based tissue regeneration is a promising approach for periodontal tissue regeneration. Previously, we generated a swine model of periodontitis, and demonstrated that transplantation of both autologous and allogeneic periodontal ligament stem cells (PDLSCs) into the surgically created periodontal defect areas in miniature pigs were capable of regenerating periodontal tissues, indicating that PDLSCs-mediated tissue engineering would be a favorable treatment for periodontitis [15-17]. However, inflamed autogenous PDLSCs had markedly dysfunctional properties [18]; moreover, the sources of PDLSCs are limited, largely impeding the clinical application of this approach. Thus, the potential of other types of MSCs for periodontal regeneration was also investigated. Besides dental pulp cells such as PDLSCs and dental pulp stem cells (DPSCs), non-dental stem cells such as bone marrow mesenchymal stem cells (BMMSCs) and adipose-derived stem cells (ADSCs) are the main stem cell sources currently used for periodontal regeneration in preclinical and clinical research on periodontitis [19]. Since periodontitis is a chronic infectious disease associated with inflammation in the regeneration microenvironment, the high expression of inflammatory products such as tumor necrosis factor-alpha (TNFα) and lipopolysaccharide (LPS) [20] can impair the biological features of MSCs, thereby inducing senescence and affecting the tissue regeneration efficiency [21]. As a result, these different types of MSCs show variable tissue regeneration efficiencies depending on their specific properties. Unfortunately, the biological properties and senescence resistance of different types of stem cells remain largely unknown. Therefore, in the present study, we compared the stemness, proliferation, and senescence rates, along with the osteogenesis properties among DPSCs, PDLSCs, BMMSCs, and ADSCs from passage three to six *in vitro*. We also compared the resistance to LPS-induced apoptosis, senescence, and osteogenesis *in vitro*; determined the underlying mechanisms and pathways through gene expression profiling and functional analyses; and evaluated the ability of injection of the different cell types for *in vivo* periodontal regeneration in a minipig model of periodontitis.

## MATERIALS AND METHODS

### Cell Culture

All human stem cells were obtained from the Department of Experimental Hematology, Beijing Institute of Radiation Medicine. BMMSCs, ADSCs, DPSCs and PDLSCs were originally extracted from iliac bone marrow, adipose tissue, dental pulp and periodontal tissue respectively, from five healthy male subjects (16-20 years old) without any diseases under approved guidelines set by the Department of Experimental Hematology, Beijing Institute of Radiation Medicine, with informed consent. Single-cell suspensions were obtained by passing the cells through a 70-μm strainer (Falcon; BD Labware, Franklin Lakes, NJ). Cells were expanded in a GMP-compliant facility with ISO 8 clean room standards equipped with class II and class III bio-safety cabinets and all other standard tissue culture equipment. The xenobiotic-free cell culture medium was used to cell culture, which is containing animal free origin collagenase (Worthington Biochemical Corporation, Lakewood, NJ), CELLstart, EZPassage Tool, HBSS-Ca/Mg free, D/F12, TrypLE, xeno-free B27, N2 supplement, MSCGM-CD and ProFreeze CDM (Invitrogen/Gibco, Carlsbad, CA), human serum (Innovative Research, Inc., Novi, Michigan), basic fibroblast growth factor-2 (bFGF-2) (Peprotech, Rocky Hill, NJ), TeSR2 which includes high levels of bFGF-2 together with transforming growth factor-β (TGF-β) (Stem Cell Technologies, Vancouver, BC, Canada), and Nutristem Stemedia (Stemgent, San Diego), which consists of human recombinant insulin, human serum albumin, transferrin, human fibroblast growth factor, and TGF-β. The same passage of BMMSCs, ADSCs, DPSCs and PDLSCs were used for each experiment, all cells used in this study were passage 3 and passage 6, which were 15-20 divisions of the primary cells.

### Cell Proliferation and Apoptosis

Single-cell suspensions (1×10^5^ cells) from five healthy male subjects were seeded on 6 well plates incubated at 37°C in 5% CO_2_. Cell proliferation was detected by collecting and counting at 1, 2, 3 days, respectively. Also, population doubling was calculated during both passage three and passage six following the formula: PD=log2 (number of collected cells/ number of seeded cells). Results were represented as mean ± standard deviation of three independent experiments performed in triplicate.

Cell apoptosis was evaluated using the FITC Annexin V Apoptosis Detection Kit I (BD Biosciences). Cells were washed twice with cold phosphate-buffered saline (PBS; Invitrogen) and centrifuged at 200 g for 5 min. The cell pellet was resuspended in 1X Binding Buffer at a concentration of 1×10^6^ cells/ml, transfer 100 µl of the solution, add 5 µl of FITC Annexin V and 5 µl PI, gently vortex the cells and incubate for 15 min at room temperature (25°C) in the dark, and analyzed by flow cytometry.

### Osteogenic Induction

To evaluate the osteogenic differentiation potential in vitro, 1×10^5^ MSCs (three and six passages) from five healthy male subjects were seeded on 6 well plates (BD Biosciences). Subcon?uent cultures were incubated in the osteogenic medium (Invitrogen) for 2 weeks. The medium was changed every 2 days. Then cells were fixed with 70% ethanol, and Alizarin Red staining was used to determine the potential of osteogenic differentiation of MSCs. The mineralization was measured by using the Image-Pro Plus 6.0 program (Media Cybernetics, Rockville, MD, USA).

### Senescence-associated β-galactosidase (SA β-gal) Staining and Quantitative Analysis

Single-cell suspensions (1×10^5^ cells) from five healthy male subjects were seeded on 6 well plates incubated at 37°C in 5% CO_2_ for 12 hours. After fixed and washed with PBS, cells were incubated with stain solution, which was freshly prepared containing 2 mg/ml 5-bromo-4-chloro-3-indolyl β-D-galactopyranoside, 40 mmol/L citric acid/sodium phosphate (pH 6.0), 5 mmol/L potassium ferrocyanide, 5 mmol/L potassium ferricyanide, 150 mmol/L NaCl, 30 mmol/L MgCl_2_, at 37 °C for 18 hours. Quantification of SA β-gal staining measured by using the Image-Pro Plus 6.0 program (Media Cybernetics, Rockville, MD, USA).

### Cellular ROS Detection Assay

Cells from five healthy male subjects were seeded onto 6-well culture plates to ensure 50-70% confluency on the day of the experiment. Load the cells with the ROS/RNS 3-Plex Detection Mix, using a volume sufficient to cover the cell monolayer and incubate under normal tissue culture conditions for 2 hours. Carefully remove the ROS/RNS 3-Plex Detection Mix from the glass slides by gently tapping them against layers of paper towel or from tissue culture plates. Carefully wash cells twice with 1X Wash Buffer in a volume sufficient to cover the cell monolayer. Overlay the cells with a cover slip and observe them under a fluorescence/confocal microscope using standard excitation/emission filter sets. Quantification measured by using the Image-Pro Plus 6.0 program (Media Cybernetics, Rockville, MD, USA).

### Total RNA Extraction and Microarray Assay

Cells (BMMSCs and DPSCs) from five healthy male subjects were seeded in 10 cm^2^ dishes and cultured until they reached 80 % confluence. Cells were then treated with VP16 for 48 h overnight. Total RNA was extracted using Trizol Reagent (Life Technologies). Following purification with a RNeasy kit (Qiagen, Valencia, CA, USA), cDNA was generated using One-Cycle Target Labeling and Control Reagents (Affymetrix, Santa Clara, CA, USA), and cRNA was created with a GeneChip IVT Labeling Kit (Affymetrix, Santa Clara, CA, USA). Biotin-labeled, fragmented (B200 nt) cRNA was hybridized for 16 h at 45 ? to Affymetrix GeneChip Human Gene 1.0 ST arrays (Affymetrix). For each sample, three biological replicates were performed. All arrays were washed, stained, and read by a GeneChip Scanner 3000 (Affymetrix). The fluorescence signal was excited at 570 nm, and data were collected on a confocal scanner. Data were analyzed by GeneChip Operating Software 1.4. Differentially expressed genes were identified based on RVM t test and false discovery rate (FDR) analysis. Differentially expressed genes with at least 1.2-fold change in either direction with P<0.05 were considered to be up or down regulated. Based on the Kyoto Encyclopedia of Genes and Genomes (KEGG) database, significantly changed pathways were identified and connected in a pathway network (Path-net) to show the relationship between these pathways. Each pathway in the network was measured by counting its number of upstream and downstream pathways, which were shown as in-degree, out-degree or degree, respectively. A higher degree of a pathway indicated that it regulated or was regulated by other pathways, implying a more important role in the signaling network.

GO analysis was used to organize differentially expressed genes into hierarchical categories. Afterwards, based on the GO and pathway analysis, an interactions repository (Signal net) derived from KEGG was established to show the core genes that played an important role in this network. The nodes in the network were connected when their corresponding encoded gene products were connected directly or indirectly by a linker gene in the interaction network. The network for each gene was measured by counting the number of upstream and downstream genes or binding genes, which were shown as in-degree, out-degree or degree, respectively. A higher degree indicated that a gene regulated or was regulated by more genes, implying a more important role in the signaling network. The gene expression data discussed in this publication have been deposited in the NCBI Gene Expression Omnibus and are accessible through GEO Series accession number GSE43498 (www.ncbi.nlm.nih.gov/geo/query/acc.cgiacc=GSE43498.acc=GSE43498).

### qRT-PCR

Applied Biosystems software was used to design optimal primer pairs for quantitative RT-PCR. Total RNA was extracted from the cells using TRIZOL (Invitrogen) and the cDNA was synthesized from 2 µg aliquots of RNA, oligo(dT), and reverse transcriptase, according to the manufacturer’s protocol (Invitrogen). Amplifications of target genes were performed by real-time quantitative PCR (qPCR) using the cDNA as template, the specific primers and QuantiTect SYBR Green PCR kit (Qiangen, Hilden, Germany) on an ABI PRISM 7900 Real Time PCR System (Applied Biosystems, Carlsbad, USA). The gene expression changes were determined by using the method of 2-△△CT. The raw quantifications were normalized to the GAPDH values for each sample and fold changes were shown as Mean ± SD in three independent experiments with each triplicate.

### LPS and TNFα Stimulation

Single-cell suspensions (1×10^5^ cells) from five healthy male humans were seeded on 6-well plate and cultured with complete medium containing 1 μg/ml LPS or 100ng/ml TNFα for 3 days. Then cells were collected and apoptosis, SA β-gal staining and osteogenesis were analyzed.

### Transplantation in Nude Mice

Approximately 4.0×10^6^ of cells were mixed with 40 mg of hydroxyapatite/tricalciumphosphate (HA/TCP) ceramic particles, combined with/without 1 μg/ml LPS and then transplanted subcutaneously into the dorsal surface of 10-week-old immunocompromised beige mice. 8 weeks after transplantation, the transplants were harvested, fixed with 10% formalin, decalcified with buffered 10% EDTA (pH 8.0), and then embedded in paraffin. 5 μm sections were obtained, deparaffinized, hydrated and stained with H&E staining. Bone formation area was measured by using the Image-Pro Plus 6.0 program (Media Cybernetics, Rockville, MD, USA).

### Generation of the Bone Defect and MSCs Injection

Six miniature pigs were used to generate periodontitis lesions of the first molars as previously reported^4^ for a total of 12 defects according to the animal care and use committee of Capital Medical University (Reference number: AEEI-2015-089). Surgical procedure and cell injection were performed accordingly as we previous study: The alveolar bone defect was 5 mm in width, 7 mm in length, and 3 mm in depth, and notch-shaped marks were made on the root surface at the level of the top of the alveolar crest and the floor of the defect. 12 defects divided into 3 groups, namely control group, BMMSCs group and DPSCs groups, each 4 defects. The BMMSCs group and DPSCs groups were separately injected with approximately 1×10^7^ human cells (from four healthy male human) in 0.6 ml of 0.9% NaCl at 3 sites (approximately 0.2 ml per site): the mesial side of the bone defect, the distal side of the bone defect, and the middle of the bone defect. The control group was injected with 0.9% NaCl at the same sites as cell injection group. At 12 weeks after transplantation, all animals were sacrificed, and samples were harvested and fixed with 4% paraformaldehyde (Sigma-Aldrich Corp) and assessed histologically.

### Radiological Evaluations

At 4 weeks after surgical creation of defect and 12 weeks after cell implantation, these defects were examined by computed tomography (CT) (Siemens, Erlangen, Germany) to monitor the defect shape. The scanning conditions were: 120 kV, 250 mA, 0.75 mm slice thickness, and 3-second slice acquisition time. Data were stored using the Dicom 3.0 standard. Three-dimensional CT imaging was reconstructed to assess the tissue regeneration. Dicom format default images were introduced into Mimics software. Threshold values were set according to the Bone (CT) Scale in Mimics. Three-dimensional models were reconstructed using Optimal, a setting in Mimics. An ASCII stereolithography (STL) file of the bone was imported into Geomagic Studio and the bone volume was measured.

**Figure 1.**
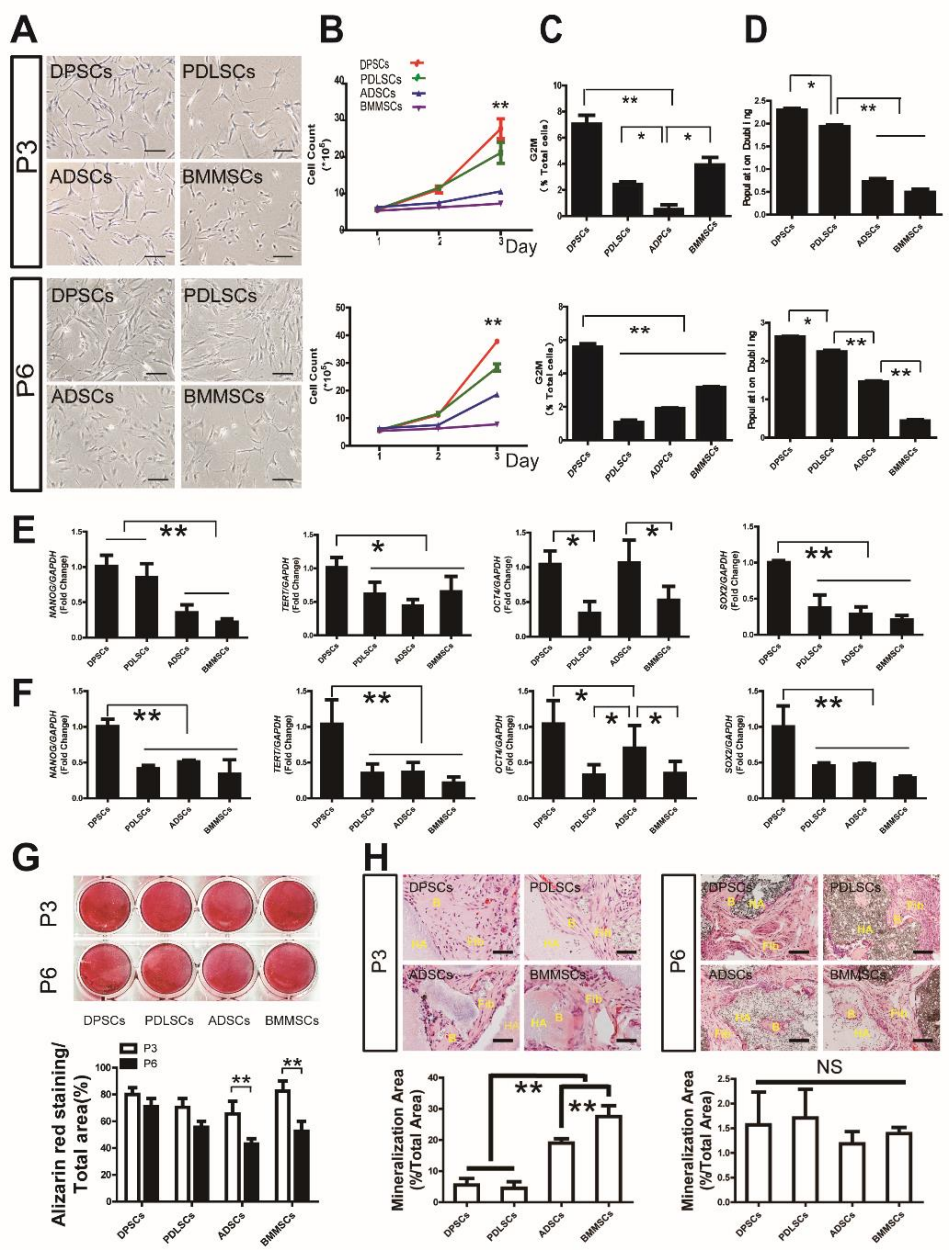
DPSCs have higher proliferation and osteogenic capability maintenance compared to PDLSCs, ADSCs, and BMMSCs during aging (**A**) Gross view of different MSCs at passages three and six. (**B**) Cell count analysis showed that both passage three and six DPSCs have a higher proliferation rate than that of other stem cells. (**C**) Cell cycle analysis also indicated more DPSCs at G2 phase compared to other stem cells at both passage three and six. (**D**) Population doubling of different MSCs at passage three and passage six showed that DPSCs have a higher PD value. (**E, F**) Real-time RT-PCR analysis revealed that *NANOG, SOX2, TERT*, and *OCT4* were expressed at higher levels in passage three and six DPSCs than in the other stem cells. (**G**) Alizarin Red staining and quantitative measurement revealed no significant difference in mineralization of DPSCs, PDLSCs, ADSCs and BMMSCs at passage three, while both ADSCs and BMMSCs of passage six exhibited declined mineralization potential and were lower compared to DPSCs and PDLSCs. (**H**) ADSCs and BMMSCs showed higher osteogenesis ability *in vivo* than DPSCs and PDLSCs of passage three, with no significant difference among groups at passage six. Values are means ± SDs. One-way ANOVA was used to determine statistical significance. Error bars represent SDs (n = 5). *P ≤ 0.05; **P ≤ 0.01; NS: no significance. Scale bar: 100 μm. B: bone-like tissue, Fib: fibrous tissue, HA: hydroxyapatite/ tricalciumphosphate.

### Quantitative and Histological Assessment of Regenerated Periodontal Tissues

At 12 weeks after transplantation, all animals were sacrificed and the samples from the experimental area were harvested and fixed with 4% formaldehyde. The heights of new bone regeneration were measured using a Williams periodontal probe: the distance from the top of newly formed bone to notch-shaped CEJ marks made during the operation was scaled. Each sample was measured at three different positions from the buccal to the lingual side. Mean values were recorded, and the heights of new bone regeneration were 7 mm minus mean values. The proportion of bone volume occupying the virtual spaces of the defect was measured, allowing quantitative comparisons among the three groups. Then, the harvested samples were subsequently decalcified with buffered 10% edetic acid (pH 8.0) for 8 to 12 weeks and embedded in paraffin. Sections were deparaffinized and stained with hematoxylin and eosin (H&E). For histopathological assessment, buccal-lingual-direction sections of the experimental region were cut. Sections (5 μm) were deparaffinized and stained with H&E. For quantification of bone formation, the extent of bone within each section was analyzed semi-quantitatively by NIH Image J as described previously, 5 representative areas at ×5 magnification in each group were used. The area of bone formation was expressed as the percentage of bone in the periodontium in the sections.

**Figure 2.**
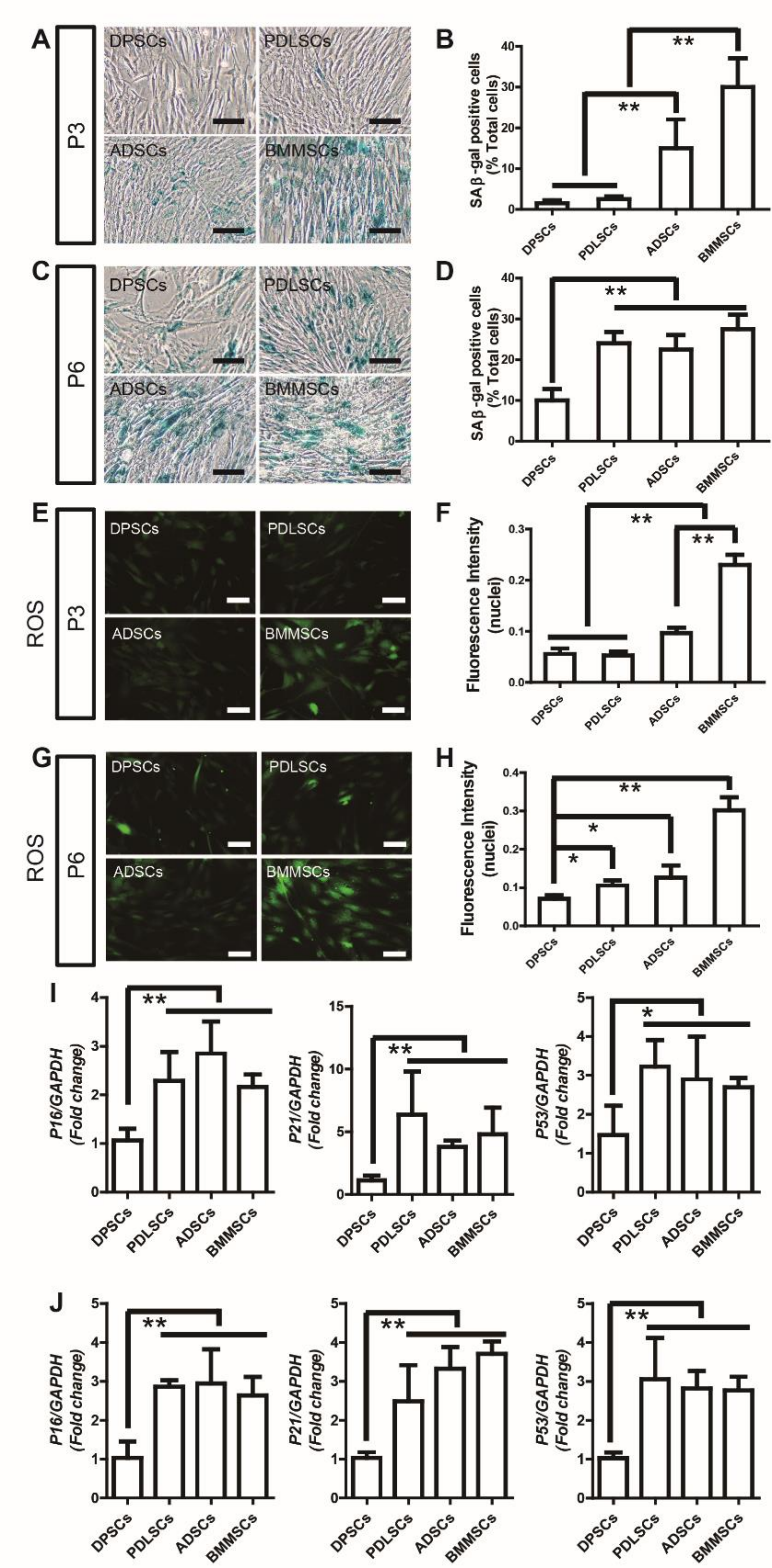
DPSCS show higher ability for senescence resistance than PDLSCs, ADSCs, and BMMSCs (**A**) Senescence-associated β-galactosidase (SA β-gal) staining of DPSCs, PDLSCs, ADSCs, and BMMSCs at passage three. (**B**) Quantitative analysis indicated significantly more SA β-gal-positive cells in passage three BMMSCs and ADSCs compared to DPSCs and PDLSCs. (**C**) SA β-gal staining of DPSCs, PDLSCs, ADSCs, and BMMSCs at passage six. (**D**) Quantitative analysis indicated significantly more SA β-gal-positive cells in passage six PDLSCs, BMMSCs, and ADSCs compared to DPSCs. (**E**) Reactive oxygen species (ROS) staining of DPSCs, PDLSCs, ADSCs, and BMMSCs at passage three. (**F**) Quantitative analysis indicated significantly lower ROS generation in passage three DPSCs and PDLSCs compared to ADSCs and BMMSCs. (**G**) ROS staining of DPSCs, PDLSCs, ADSCs, and BMMSCs at passage six. (**H**) Quantitative analysis indicated significantly lower ROS generation in passage six DPSCs compared to PDLSCs, ADSCs, and BMMSCs. (**I**) Real-time RT-PCR analysis revealed that theP16, P21, P53 were downregulated in DPSCs compared to other MSCs at passage three. (**J**) Real-time RT-PCR analysis revealed that theP16, P21, P53 were downregulated in DPSCs compared to other MSCs at passage six. Values are means ± SDs. One-way ANOVA was used to determine statistical significance. Error bars represent SDs (n = 5). *P ≤ 0.05; **P ≤ 0.01. Scale bar: 100 μm.

### Statistical analysis

Each experiment was done independently at least three times with similar results. Results are expressed as mean ± SD. Significant differences were assessed with one-way ANOVA (two-tailed). P<0.05 was considered to be statistically significant.

## RESULTS

### DPSCs Maintain Higher Proliferation and Osteogenesis Capability Compared to PDLSCs, ADSCs, and BMMSCs during Aging

The proliferation and multi-differentiation ability of stem cells are important properties for applications in stem cell-based medicine. To obtain a more abundant source of stem cells for therapeutic efficacy, the cells need to be sub-cultured for several passages. However, the proliferation and multi-differentiation ability can decline during aging. For practical application, MSCs cultured to passage six would be sufficiently abundant for tissue engineering; in addition, MSCs cultured to passage six would have a senescence phenotype that would be valuable for research. Therefore, the proliferation and osteogenic ability of DPSCs, PDLSCs, ADSCs, and BMMSCs were compared at passages three and six. Cell count analysis showed that both passage three and passage six DPSCs had higher proliferation rates than the other stem cell types (Fig. 1A, B). Cell cycle analysis also indicated that there were more DPSCs at the G2 phase compared to the other stem cells at both passage three and passage six (Fig. 1C, Supplemental Fig. 1). In addition, the degree of population doubling (PD) was compared among the stem cell types. Again, the DPSCs showed the highest PD compared to the other cell types at both passage three and six (Fig. 1D). We further examined the expression of key genes associated with self-renewal ability. Real-time reverse transcription-polymerase chain reaction (RT-PCR) analysis revealed that the expression levels of *NANOG*, *SOX2*, *TERT*, and *OCT4* were higher in both passage three and six DPSCs than those of the other stem cells (Fig. 1E, F).

Moreover, two weeks after induction in osteogenic medium, Alizarin Red staining and quantitative measurement revealed no significant difference in the mineralization of DPSCs, PDLSCs, ADSCs, and BMMSCs at passage three, whereas both ADSCs and BMMSCs of passage six exhibited declined mineralization potential, which was lower compared to that of DPSCs and PDLSCs (Fig. 1G).

The *in vivo* data also showed that ADSCs and BMMSCs had higher osteogenesis ability than DPSCs and PDLSCs at passage three, whereas there was no significant difference in the osteogenic ability of the tissues derived from passage six DPSCs, PDLSCs, ADSCs, and BMMSCs (Fig. 1H).

**Table 1:** Top ten down-regulated genes ranked by degree after analysis of Signal-net. (BMMSCs Vs. DPSCs).

Gene symbol	Degree	Indegree	Outdegree	Gene feature
ENTPD1	112	56	56	Down
PLCB4	83	50	33	Down
PIK3R1	81	60	21	Down
MAPK1	76	40	36	Down
ACTB	75	47	28	Down
ACTG1	75	47	28	Down
PIK3CB	71	55	16	Down
NME1	66	33	33	Down
CANT1	62	31	31	Down
NT5C2	62	31	31	Down

BMMSCs, bone marrow mesenchymal stem cells. DPSCs, dental pulp stem cells.

### DPSCs Show Enhanced Senescence Resistance Compared to PDLSCs, ADSCs, and BMMSCs

Senescence-associated β-galactosidase (SA β-gal) and reactive oxygen species (ROS) staining were used to detect the senescence of the stem cells cultured from passage three to six. As shown in Fig. 2, quantitative analysis indicated significantly more SA β-gal- and ROS-positive cells in passage three BMMSCs and ADSCs compared to DPSCs and PDLSCs. Moreover, the numbers of SA β-gal- and ROS-positive cells increased along with aging to passage six in all stem cells. However, passage six BMMSCs, ADSCs, and PDLSCs presented more SA β-gal- and ROS-positive cells than DPSCs. These results suggested that DPSCs have a superior cell senescence-resistant property. RT-PCR analysis revealed that the expression levels of p53, p21, and p16 were lower in DPSCs of passage three and six compared to those of the other MSCs (Fig. 2I, J).

### DPSCs and BMMSCs Differ in Mitogen-activated Protein Kinase (MAPK) Signaling Pathway and Apoptosis Expression Profiles

**Figure 3.**
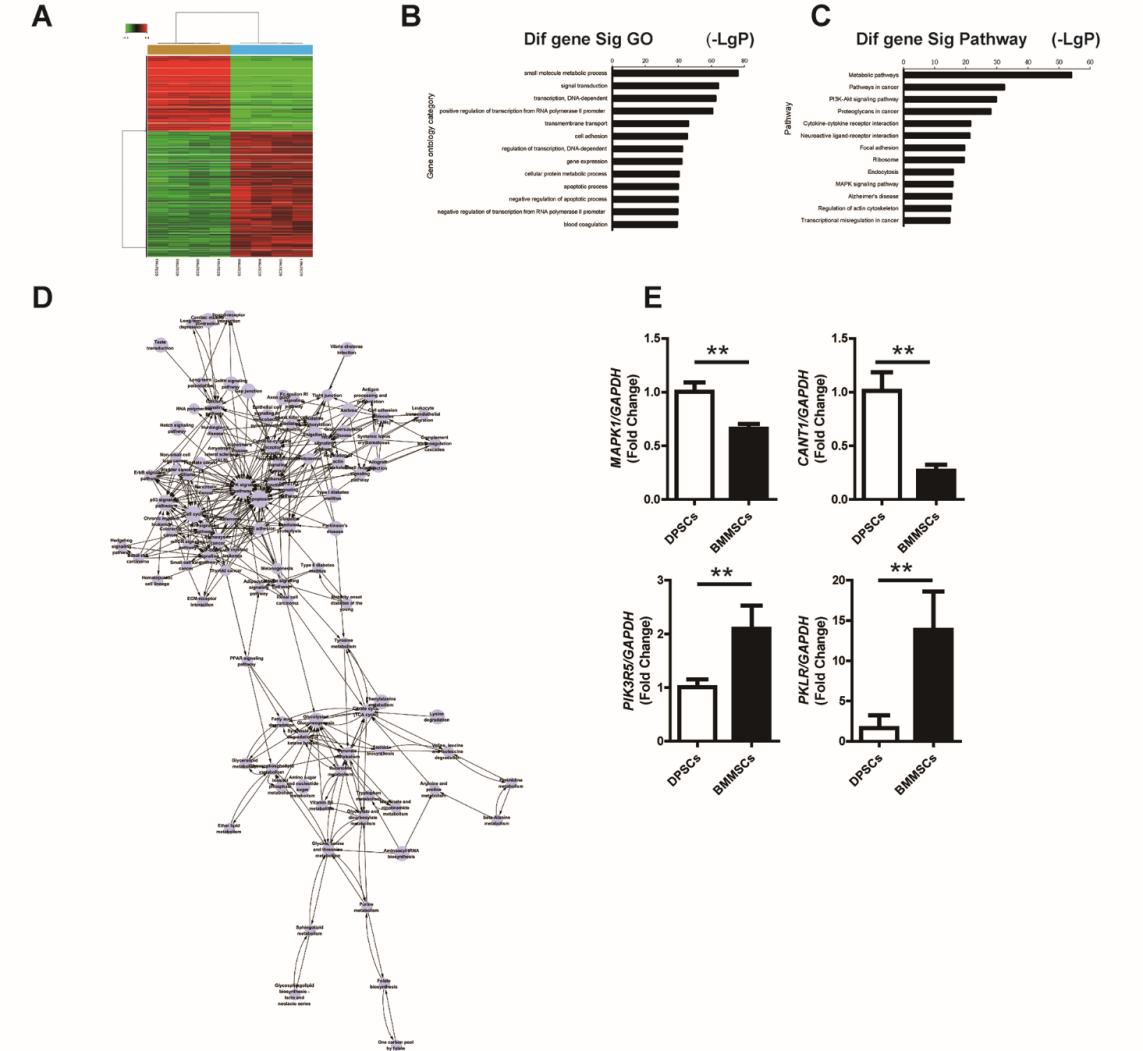
DPSCs have reduced activation of the MAPK signaling pathway and a lower apoptosis expression profile compared to BMMSCs (**A**) Affymetrix GeneChip Human Gene 1.0 ST arrays analysis of BMMSCs and DPSCs. (**B**) The top 13 GO terms for the differentially expressed genes (-LgP > 28) between BMMSCs and DPSCs. (**C**) The top 13 pathways enriched for differentially expressed genes between BMMSCs and DPSCs. (**D**) Path-net analysis of the interaction network covering the significantly changed pathways between DPSCs and BMMSCs. (**E**) Real-time RT-PCR analysis revealed that the *MAPK1* and *CANT1* level were decreased, and the *PIK3R5* and *PKLR* levels were increased in BMMSCs at passage three compared to DPSCs. *GAPDH* was used as an internal control. Values are means ± SDs. Student’s t-tests were used to determine statistical significance. Error bars represent SDs (n = 5). **P ≤ 0.01

As the results described above indicated that DPSCs have the highest senescence-resistant property, while BMMSCs have the lowest senescence-resistant property, to further reveal the mechanism of the senescence-resistant property of MSCs, a gene expression profiling approach was used to compare the functional gene expression changes in BMMSCs and DPSCs with Affymetrix GeneChip Human Gene 1.0 ST arrays analysis. Overall, 9916 differentially expressed genes were detected between the two types of stem cells, 6188 of which were up-regulated and 3728 were down-regulated (Fig. 3A); the top 10 up- and downregulated genes in BMMSCs compared to DPSCs are listed in Table 1 and Table 2, respectively. The differentially expressed genes were then subjected to Gene Ontology (GO) analysis, and the top GO terms (-LgP > 28) associated with these genes were found to be response to small-molecule metabolic process, signal transduction, transcription (DNA-dependent), positive regulation of transcription from RNA polymerase II promoter, transmembrane transport, cell adhesion, regulation of transcription (DNA-dependent), gene expression, cellular protein metabolic process, apoptotic process, negative regulation of apoptotic process, negative regulation of transcription from RNA polymerase II promoter, blood coagulation, multicellular organismal development, proteolysis, synaptic transmission, translation, innate immune response, and positive regulation of transcription (DNA-dependent) (Fig. 3B). Therefore, the GO analysis clearly showed that many of the genes that are differentially expressed between DPSCs and BMMSCs regulate important functions for self-renewal ability and metabolic processes, including apoptosis, cell cycle, gene expression, and cellular protein metabolic process.

**Table 2:** Top ten up-regulated genes ranked by degree after analysis of Signal-net. (BMMSCs Vs. DPSCs).

Gene symbol	Degree	Indegree	Outdegree	Gene feature
ENTPD3	112	56	56	Up
ENTPD8	112	56	56	Up
PIK3R5	85	64	21	Up
PKLR	82	42	40	Up
PRKACG	81	11	70	Up
ADCY8	76	45	31	Up
ADCY5	73	43	30	Up
ADCY4	72	42	30	Up
PLCG2	71	37	34	Up
NME3	66	33	33	Up

BMMSCs, bone marrow mesenchymal stem cells. DPSCs, dental pulp stem cells.

The top Kyoto Encyclopedia of Genes and Genomes (KEGG) pathways (Supplemental Fig. 2) enriched for the differentially expressed genes included metabolic pathways, pathways in cancer, PI3K-AKT signaling pathway, proteoglycans in cancer, cytokine-cytokine receptor interaction, neuroactive ligand-receptor interaction, focal adhesion, ribosome, endocytosis, MAPK signaling pathway, Alzheimer's disease, regulation of actin cytoskeleton, transcriptional mis regulation in cancer, JAK-STAT signaling pathway, ECM-receptor interaction, phagosome, dilated cardiomyopathy, protein digestion and absorption, calcium signaling pathway, and Hippo signaling pathway (Fig. 3C). We performed Path-net analysis to generate an interaction network covering these significantly changed pathways between DPSCs and BMMSCs. MAPK signaling, and apoptosis showed the highest degrees of connection in the network, suggesting that these two pathways play a core role in conferring DPSCs with the observed biological properties (Fig. 3D). These results were confirmed with real-time RT-PCR analysis, demonstrating that the expression levels of *MAPK1* and *CANT1* were decreased, and the levels of *PIK3R5* and *PKLR* were increased in BMMSCs at passage three compared to those of DPSCs (Fig. 3E).

### DPSCs Show Greater Resistance to LPS-induced Senescence and Dysfunction of Osteogenesis Compared to BMMSCs

**Figure 4.**
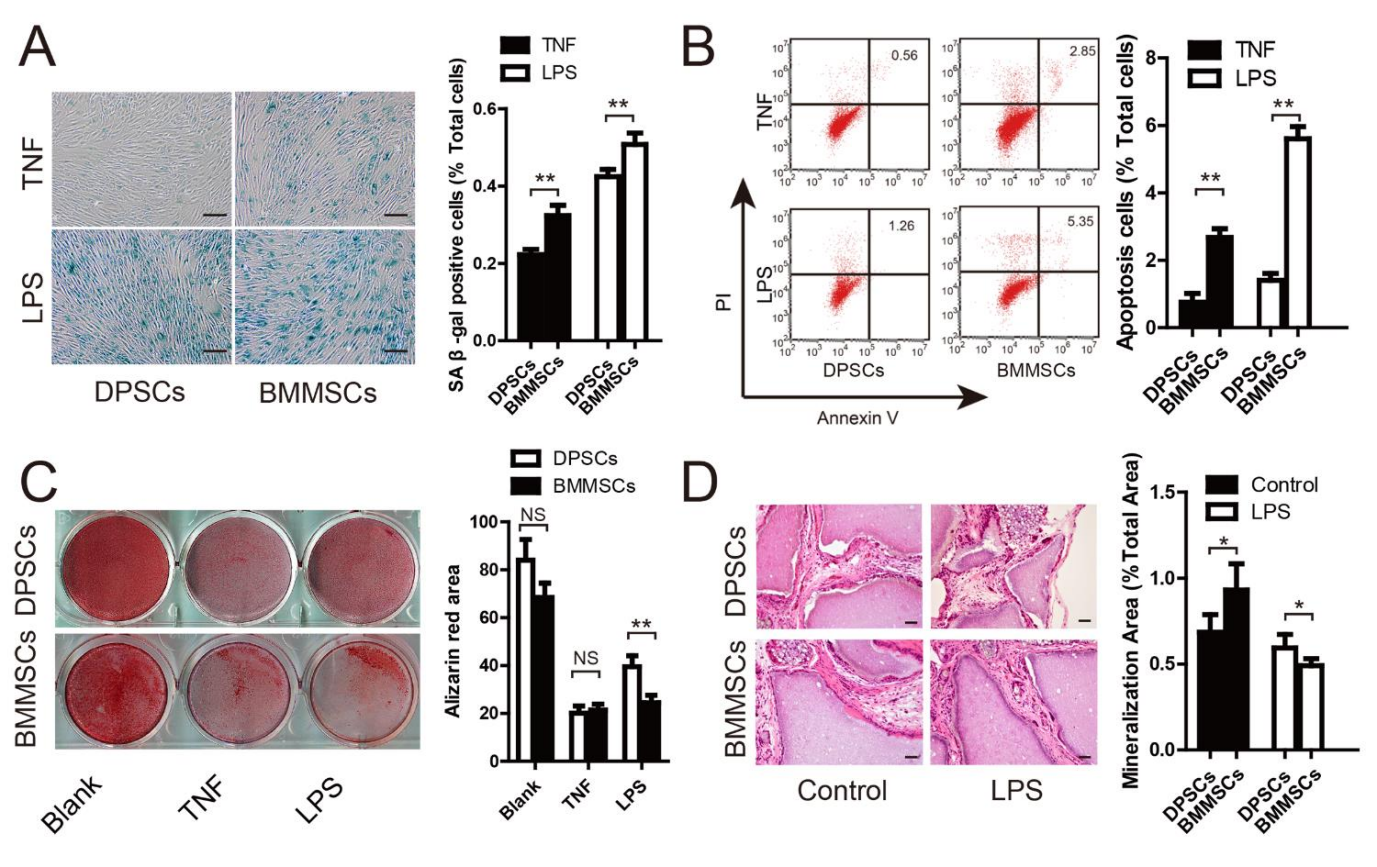
DPSCs show higher resistance to LPS-induced senescence and dysfunction of osteogenesis compared to BMMSCs (**A**) SA β-gal staining of DPSCs and BMMSCs under TNFα and LPS stimulation. Quantitative analysis indicated significantly more SA β-gal-positive cells in BMMSCs compared to DPSCs under TNFα and LPS stimulation. (**B**) Annexin V/PI staining and quantitative analysis showed lower rates of cell apoptosis in DPSCs than BMMSCs under LPS and TNFα stimulation. (**C**) Alizarin Red staining and quantitative measurements of osteogenesis of DPSCs and BMMSCs under LPS and TNFα stimulation. (**D**) HE staining and quantitative measurements of the osteogenic property of DPSCs and BMMSCs *in vivo* under LPS and TNFα stimulation. Values are means ± SDs. Student’s t-tests were used to determine statistical significance. Error bars represent SDs (n = 5). *P ≤ 0.05; **P ≤ 0.01; NS: no significance. Scale bar: 100 μm.

As a chronic inflammation disease, periodontitis is associated with dysfunction of MSCs, and the high levels of LPS and TNFα in the periodontal environment could influence the efficiency of MSCs-based tissue regeneration. Therefore, to verify the superiority of DPSCs to BMMSCs in an inflammatory environment, the cells were stimulated with 100 ng/ml TNFα and 1 μg/ml LPS, and then apoptosis, senescence, and osteogenesis were evaluated. SA β-gal staining showed that TNFα and LPS could induce cell senescence, although a lower proportion of SA β-gal-positive cells was found in DPSCs than BMMSCs (Fig. 4A). Cell cycle analysis indicated no significant difference in DPSCs and BMMSCs under LPS and TNFα stimulation (Supplemental Fig. 3), whereas Annexin V and propidium iodide staining showed a lower rate of apoptosis in DPSCs than in BMMSCs under LPS and TNFα stimulation (Fig. 4B). Alizarin Red staining and quantitative measurements revealed that inflammation could largely inhibit the osteogenesis of both DPSCs and BMMSCs (Fig. 4C). Although there was no significant difference in osteogenesis between the two stem cell types under TNFα stimulation, DPSCs demonstrated greater osteogenesis ability than BMMSCs under LPS stimulation. In addition, a greater amount of mineral tissues formed in periodontal tissues derived from transplanted DPSCs compared to BMMSCs in nude mice with LPS treatment (Fig. 4D).

### Hard Tissue Regeneration in the Periodontium Improved After DPSCs Injection Compared to BMMSCs Injection

**Figure 5.**
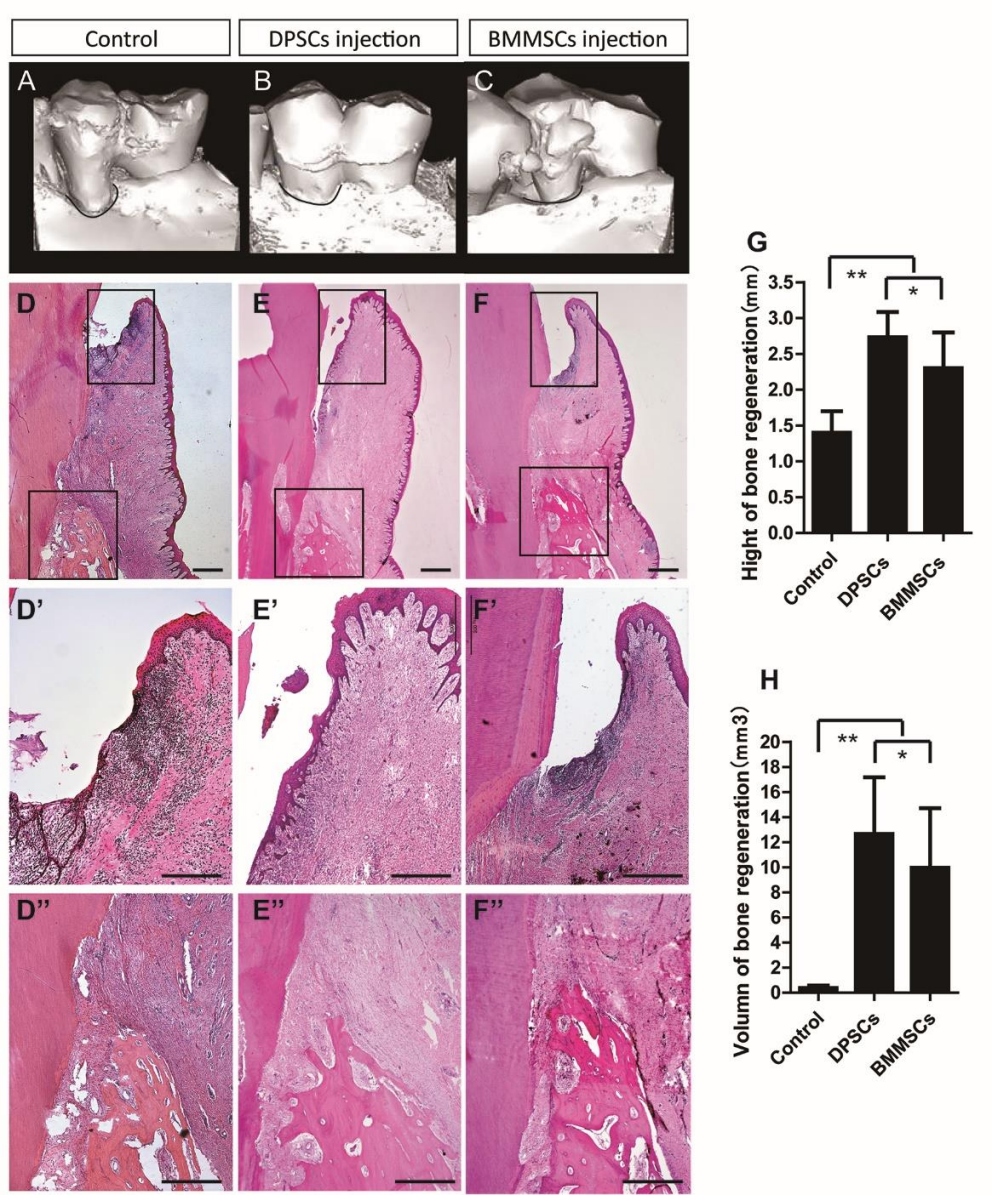
More hard tissue regeneration in the periodontium after DPSCs injection compared to BMMSCs injection *in vivo* (**A-C**) Gross micro-CT view of periodontal defect regeneration in the control group (saline injection), DPSC group, and BMMSC group. (**D-F**) Histopathological assessment of periodontal bone regeneration by HE staining in the periodontal defects of the DPSCs group and BMMSCs group. (**D’-F’**) Impaired sulcular epithelia and lymphocyte infiltration were evident in the control group compared with the DPSCs and BMMSCs groups. (**D’’-F’’, G, H**) Alveolar bone heights in the DPSCs and BMMSCs groups were much larger than those of the control group. Values are means ± SDs. One-way ANOVA was used to determine statistical significance. Error bars represent SDs (n = 5). *P ≤ 0.05; **P ≤ 0.01. Scale bar: 100 μm.

Three-dimensional computed tomography (CT) images indicated marked bone regeneration in both the DPSC and BMMSC injection groups after cell transplantation, whereas limited bone formation was detected in the control group (Fig. 5A-C). At 12 weeks post-transplantation, statistical analysis indicated that both MSC injection treatments significantly improved periodontal hard tissue regeneration in comparison with that of the control group (Fig. 5D-F). The heights and volume of new bone regeneration were also significantly higher in the MSC injection groups than those of control group (Fig. 5D”-F”). The height of periodontal alveolar bone was higher in the DPSC injection group (2.72 ± 0.33 mm) than that in the BMMSC injection group (2.28 ± 0.48 mm), and both were significantly greater than that of the control group (1.43 ± 0.41 mm). The CT scan and three-dimensional CT imaging showed that the volume of regenerative alveolar bone in the DPSC and BMMSC injection group was 12.83 ± 4.41 mm^3^ and 12.83 ± 4.34 mm^3^, respectively, which were significantly larger than that of the control group (0.58 ± 0.32 mm^3^). Impaired sulcular epithelia and lymphocyte infiltration were evident in the control group but not in the DPSCs and BMMSCs groups (Fig. 5D'-F'). Notably, the periodontal bone regeneration capacity was significantly greater for the DPSC injection group than for the BMMSC injection group.

### DISCUSSION

We demonstrated that DPSCs had superior ability to maintain their stem cell properties during aging (culture from passage three to six) compared to other MSCs, with a markedly greater proliferation rate, higher stemness, lower cellular senescence, and better osteogenic property. These superior properties of DPSCs were attributed to their distinct gene expression profile, including genes related to apoptosis, cell cycle, and metabolic processes. In addition, DPSCs were superior in resisting LPS-induced apoptosis, senescence, and osteogenesis dysfunction both *in vitro* and *in vivo*, and resulted in more effective periodontal regeneration *in vivo* in the periodontitis minipig model. Together, these results suggest that DPSCs have greater resistance under conditions of inflammation-induced senescence and would be a suitable stem cell source for tissue engineering under inflammation.

*Ex vivo* expansion or exposure to inflammation has been shown to be associated with the senescence of MSCs [22]. Stem cell senescence is a complex phenotype that entails changes in both function and replicative capacity, characterized by an enlarged, flattened morphology and irreversible G1 growth arrest. It has been speculated that senescence may lead to arrested regeneration in tissues, ultimately resulting in organ failure and death [23]. It is now well known that stem cells isolated from the bone marrow (BMMSCs), adipose tissue (ADSCs), and dental tissues (DPSCs, PDLSCs, etc.) have the capacity to form mineralized tissue [24, 25]. Although a few studies have compared the capacity of different stem cell types in tissue engineering, including ADSCs, BMMSCs, and DPSCs, their ability to maintain function during aging has not yet been fully explored. However, the chondrogenic potential of DPSCs has been reported to be weak, and both DPSCs and stem cells from the apical papilla showed weaker adipogenesis in comparison with BMMSCs [26]. Conversely, the osteogenesis and neurogenicity of dental stem cells were found to be more potent than those of BMMSCs [27, 28].

Although human MSCs (hMSCs) are available from different tissues, their quantity after primary culture is limited. Generally speaking, most cell therapy protocols require a minimum of 20-100 million hMSCs per treatment (autologous transplantation), suggesting that hMSCs need to be expanded *in vitro* for at least 4-8 weeks prior to transplantation [23]. Periodontitis is a primarily oral bacterial infection disease invading the supporting structures of the teeth and is characterized by tissue inflammation and destruction that eventually lead to tooth loss. The proinflammatory cytokines produced during tissue regeneration play a central role in premature cellular senescence, which occurs from cell culture to regeneration *in vivo*. Thus, MSCs with higher resistance to cellular senescence are highly desired to improve tissue regeneration; however, the cellular senescence properties of different stem cells have thus far remained unclear.

Here, we provide the first demonstration that DPSCs exhibit superior cellular senescence resistance during cell culture and under LPS stimulation. We further verified these beneficial effects of DPSCs over BMMSCs in MSCs-mediated periodontal regeneration *in vivo* in a miniature pig periodontitis model. Gene array and path-net analysis revealed that the MAPK signaling pathway and apoptosis pathway were most strongly connected in the gene network comparing the expression profiles of DPSCs and BMMSC, suggesting that these two pathways play a core role in the observed DPSC properties such as the higher proliferation rate and self-renewal potential. The MAPKs are intracellular serine/threonine protein kinases, including extracellular signal-regulated kinase1/2, p38 MAPKs, and c-Jun NH2-terminal kinase, which play important roles in diverse cellular processes such as cell growth, proliferation, differentiation, cell death, and immune responses. We showed that DPSCs, which have been explored for their potential ability of inducing periodontal regeneration in periodontitis [29, 30], have remarkable downregulated *MAPK1* gene expression compared to BMMSCs. The activated MAPKs lead to the upregulation of ROS, which is an important factor contributing to MSCs senescence. ROS activation plays a key role in cellular senescence and apoptosis by causing damage to DNA, proteins, and lipids [31]. Elevation of ROS levels can induce hematopoietic stem cell senescence through phosphorylation of p38 MAPK [32]. LPS secreted by *Porphyromonas gingivalis*, the most prominent periodontal pathogen, triggers the onset and duration of chronic periodontitis [33, 34] and can activate the recruitment of inflammatory cells, leading to the generation of prostanoids and cytokines, elaboration of lytic enzymes, and osteoclast activation, to ultimately initiate alveolar bone resorption [35]. Accordingly, localized LPS injection has been used to create models of periodontitis [36]. LPS can also increase the superoxide concentration, and the levels and expression of ROS and the pro-inflammatory cytokines prostaglandin E2, receptor activator of nuclear factor-kappa B ligand, interleukin-1, and TNF-α [37]. Various inflammation-related diseases, including atherosclerosis [38], rheumatoid arthritis [39], and pulmonary fibrosis [40], are related to the over-expression of these pro-inflammatory cytokines. LPS-activated MAPKs lead to the upregulation of ROS [41]. In addition, we found lower accumulation of intracellular ROS after DPSCs sub-culturing from passage three to passage six. Although LPS stimulation dramatically increased senescence and inhibited the osteogenesis ability of BMMSCs, it had no significant effect on these properties of DPSCs. Overall, these results suggest that the distinct expression of MAPKs confer DPSCs with superior ability for periodontal regeneration, demonstrating their suitability for stem cell therapy in periodontitis.

In conclusion, our study demonstrates a previously unrecognized senescence property of different stem cell types during cell culture *in vitro* and tissue regeneration *in vivo*, indicating that DPSCs are desirable seed cells in tissue engineering under an inflammatory microenvironment. Moreover, the sustainable ability of DPSCs suggests a promising future for further investigation and application in disease prevention and treatment.

## Supplemental data

Supplemental data are available at www.aginganddisease.org/EN/10.14336/AD.2018.0729.



## References

[1] Mundra V, Gerling IC, Mahato RI (2013). Mesenchymal stem cell-based therapy. Mol Pharm, 10: 77-89. 23215004 10.1021/mp3005148 PMC3549356

[2] Zhang H, Sun F, Wang J, Xie L, Yang C, Pan M, et al (2017). Combining Injectable Plasma Scaffold with Mesenchymal Stem/Stromal Cells for Repairing Infarct Cavity after Ischemic Stroke. Aging Dis, 8: 203-214. 28400986 10.14336/AD.2017.0305 PMC5362179

[3] Watt FM, Hogan BL (2000). Out of Eden: stem cells and their niches. Science, 287: 1427-1430. 10688781 10.1126/science.287.5457.1427

[4] Goodell MA, Rando TA (2015). Stem cells and healthy aging. Science, 350: 1199-1204. 26785478 10.1126/science.aab3388

[5] Goyns MH (2002). Genes, telomeres and mammalian ageing. Mech Ageing Dev, 123: 791-799. 11869736 10.1016/s0047-6374(01)00424-9

[6] Li Y, Wu Q, Wang Y, Li L, Bu H, Bao J (2017). Senescence of mesenchymal stem cells (Review). Int J Mol Med, 39: 775-782. 28290609 10.3892/ijmm.2017.2912

[7] Smith JA, Daniel R (2012). Stem cells and aging: a chicken-or-the-egg issue? Aging Dis, 3: 260-268. 22724084 PMC3375082

[8] Digirolamo CM, Stokes D, Colter D, Phinney DG, Class R, Prockop DJ (1999). Propagation and senescence of human marrow stromal cells in culture: a simple colony-forming assay identifies samples with the greatest potential to propagate and differentiate. Br J Haematol, 107: 275-281. 10583212 10.1046/j.1365-2141.1999.01715.x

[9] Campisi J, d'Adda di Fagagna F (2007). Cellular senescence: when bad things happen to good cells. Nat Rev Mol Cell Biol, 8: 729-740. 17667954 10.1038/nrm2233

[10] Sui BD, Hu CH, Zheng CX, Jin Y (2016). Microenvironmental Views on Mesenchymal Stem Cell Differentiation in Aging. J Dent Res, 95: 1333-1340. 27302881 10.1177/0022034516653589

[11] Pihlstrom BL, Michalowicz BS, Johnson NW (2005). Periodontal diseases. The Lancet, 366: 1809-1820. 10.1016/S0140-6736(05)67728-8 16298220

[12] Albandar JM (2007). Periodontal disease surveillance. J Periodontol, 78: 1179-1181. 17608570 10.1902/jop.2007.070166

[13] Aichelmann-Reidy ME, Reynolds MA (2008). Predictability of clinical outcomes following regenerative therapy in intrabony defects. J Periodontol, 79: 387-393. 18315419 10.1902/jop.2008.060521

[14] Liu O, Xu J, Ding G, Liu D, Fan Z, Zhang C, et al (2013). Periodontal ligament stem cells regulate B lymphocyte function via programmed cell death protein 1. Stem Cells, 31: 1371-1382. 23553748 10.1002/stem.1387

[15] Ding G, Liu Y, Wang W, Wei F, Liu D, Fan Z, et al (2010). Allogeneic periodontal ligament stem cell therapy for periodontitis in swine. Stem Cells, 28: 1829-1838. 20979138 10.1002/stem.512 PMC2996858

[16] Hynes K, Menicanin D, Gronthos S, Bartold PM (2012). Clinical utility of stem cells for periodontal regeneration. Periodontol 2000, 59: 203-227. 10.1111/j.1600-0757.2012.00443.x 22507067

[17] Liu Y, Zheng Y, Ding G, Fang D, Zhang C, Bartold PM, et al (2008). Periodontal ligament stem cell-mediated treatment for periodontitis in miniature swine. Stem Cells, 26: 1065-1073. 18238856 10.1634/stemcells.2007-0734 PMC2653213

[18] Liu D, Xu J, Liu O, Fan Z, Liu Y, Wang F, et al (2012). Mesenchymal stem cells derived from inflamed periodontal ligaments exhibit impaired immunomodulation. J Clin Periodontol, 39: 1174-1182. 23005571 10.1111/jcpe.12009

[19] Monsarrat P, Vergnes JN, Nabet C, Sixou M, Snead ML, Planat-Benard V, et al (2014). Concise review: mesenchymal stromal cells used for periodontal regeneration: a systematic review. Stem Cells Transl Med, 3: 768-774. 24744392 10.5966/sctm.2013-0183 PMC4039455

[20] Ling MR, Chapple IL, Matthews JB (2015). Peripheral blood neutrophil cytokine hyper-reactivity in chronic periodontitis. Innate Immun, 21: 714-725. 26055820 10.1177/1753425915589387

[21] Abdelmagid SM, Barbe MF, Safadi FF (2015). Role of inflammation in the aging bones. Life Sci, 123: 25-34. 25510309 10.1016/j.lfs.2014.11.011

[22] Jones DL, Rando TA (2011). Emerging models and paradigms for stem cell ageing. Nat Cell Biol, 13: 506-512. 21540846 10.1038/ncb0511-506 PMC3257978

[23] Sepulveda JC, Tome M, Fernandez ME, Delgado M, Campisi J, Bernad A, et al (2014). Cell senescence abrogates the therapeutic potential of human mesenchymal stem cells in the lethal endotoxemia model. Stem Cells, 32: 1865-1877. 24496748 10.1002/stem.1654 PMC4209016

[24] Fu X, Jin L, Ma P, Fan Z, Wang S (2014). Allogeneic stem cells from deciduous teeth in treatment for periodontitis in miniature swine. J Periodontol, 85: 845-851. 24001042 10.1902/jop.2013.130254

[25] Liu Y, Hu J, Wang S (2014). Mesenchymal stem cell-mediated treatment of oral diseases. Histol Histopathol, 29: 1007-1015. 24638842 10.14670/HH-29.1007

[26] Zhang W, Walboomers XF, Shi S, Fan M, Jansen JA (2006). Multilineage differentiation potential of stem cells derived from human dental pulp after cryopreservation. Tissue Eng, 12: 2813-2823. 17518650 10.1089/ten.2006.12.2813

[27] Davies OG, Cooper PR, Shelton RM, Smith AJ, Scheven BA (2015). A comparison of the in vitro mineralisation and dentinogenic potential of mesenchymal stem cells derived from adipose tissue, bone marrow and dental pulp. J Bone Miner Metab, 33: 371-382. 24997523 10.1007/s00774-014-0601-y

[28] Huang GT, Gronthos S, Shi S (2009). Mesenchymal stem cells derived from dental tissues vs. those from other sources: their biology and role in regenerative medicine. J Dent Res, 88: 792-806. 19767575 10.1177/0022034509340867 PMC2830488

[29] Cao Y, Liu Z, Xie Y, Hu J, Wang H, Fan Z, et al (2015). Adenovirus-mediated transfer of hepatocyte growth factor gene to human dental pulp stem cells under good manufacturing practice improves their potential for periodontal regeneration in swine. Stem Cell Res Ther, 6: 249. 26670567 10.1186/s13287-015-0244-5 PMC4681125

[30] Hu J, Cao Y, Xie Y, Wang H, Fan Z, Wang J, et al (2016). Periodontal regeneration in swine after cell injection and cell sheet transplantation of human dental pulp stem cells following good manufacturing practice. Stem Cell Res Ther, 7: 130. 27613503 10.1186/s13287-016-0362-8 PMC5017121

[31] Valko M, Leibfritz D, Moncol J, Cronin MT, Mazur M, Telser J (2007). Free radicals and antioxidants in normal physiological functions and human disease. Int J Biochem Cell Biol, 39: 44-84. 16978905 10.1016/j.biocel.2006.07.001

[32] Ito K, Hirao A, Arai F, Takubo K, Matsuoka S, Miyamoto K, et al (2006). Reactive oxygen species act through p38 MAPK to limit the lifespan of hematopoietic stem cells. Nat Med, 12: 446-451. 16565722 10.1038/nm1388

[33] Socransky SS, Smith C, Haffajee AD (2002). Subgingival microbial profiles in refractory periodontal disease. J Clin Periodontol, 29: 260-268. 11940147 10.1034/j.1600-051x.2002.290313.x

[34] Takeuchi Y, Umeda M, Sakamoto M, Benno Y, Huang Y, Ishikawa I (2001). Treponema socranskii, Treponema denticola, and Porphyromonas gingivalis are associated with severity of periodontal tissue destruction. J Periodontol, 72: 1354-1363. 11699477 10.1902/jop.2001.72.10.1354

[35] Genco CA, Van Dyke T, Amar S (1998). Animal models for Porphyromonas gingivalis-mediated periodontal disease. Trends Microbiol, 6: 444-449. 9846362 10.1016/s0966-842x(98)01363-8

[36] Dumitrescu AL, Abd-El-Aleem S, Morales-Aza B, Donaldson LF (2004). A model of periodontitis in the rat: effect of lipopolysaccharide on bone resorption, osteoclast activity, and local peptidergic innervation. J Clin Periodontol, 31: 596-603. 15257734 10.1111/j.1600-051X.2004.00528.x

[37] Roux S, Orcel P (2000). Bone loss. Factors that regulate osteoclast differentiation: an update. Arthritis Res, 2: 451-456. 11094458 10.1186/ar127 PMC128874

[38] Libby P, Ridker PM, Maseri A (2002). Inflammation and atherosclerosis. Circulation, 105: 1135-1143. 11877368 10.1161/hc0902.104353

[39] Manzi S, Wasko MC (2000). Inflammation-mediated rheumatic diseases and atherosclerosis. Ann Rheum Dis, 59: 321-325. 10784507 10.1136/ard.59.5.321 PMC1753135

[40] Coker RK, Laurent GJ (1998). Pulmonary fibrosis: cytokines in the balance. European Respiratory Journal, 11: 1218-1221. 9657557 10.1183/09031936.98.11061218

[41] Pan X, Cao X, Li N, Xu Y, Wu Q, Bai J, et al (2014). Forsythin inhibits lipopolysaccharide-induced inflammation by suppressing JAK-STAT and p38 MAPK signalings and ROS production. Inflamm Res, 63: 597-608. 24691777 10.1007/s00011-014-0731-7

