# Supplementary material for: Maintained Properties of Aged Dental Pulp Stem Cells for Superior Periodontal Tissue Regeneration
Source: Aging Dis. 2019 Aug 1;10(4):793–806. doi: 10.14336/AD.2018.0729 (PMC6675537; doi:10.14336/AD.2018.0729)
Supplement: Supplementary file 1 [file AD-10-4-793-s.pdf]

SUPPLEMENTARY DATA

Original Article

Maintained Properties of Aged Dental Pulp Stem Cells for Superior Periodontal Tissue Regeneration

Linsha Ma[1,#], Jingchao Hu[1,#], Yu Cao[1], Yilin Xie[1], Hua Wang[2], Zhipeng Fan[1], Chunmei Zhang[1], Jinsong Wang[1,3], Chu-Tse Wu[2,*], Songlin Wang[1,3,*]

[1]Molecular Laboratory for Gene Therapy & Tooth Regeneration, Beijing Key Laboratory of Tooth Regeneration and Function Reconstruction, Capital Medical University, School of Stomatology, Beijing, China
[2]Department of Experimental Hematology, Beijing Institute of Radiation Medicine, Beijing, China
[3]Department of Biochemistry and Molecular Biology, Capital Medical University School of Basic Medical Sciences, Beijing, China

© 2018. Ma L et al. Published online at http://www.aginganddisease.org/EN/10.14336/AD.2018.0728

SUPPLEMENTARY DATA

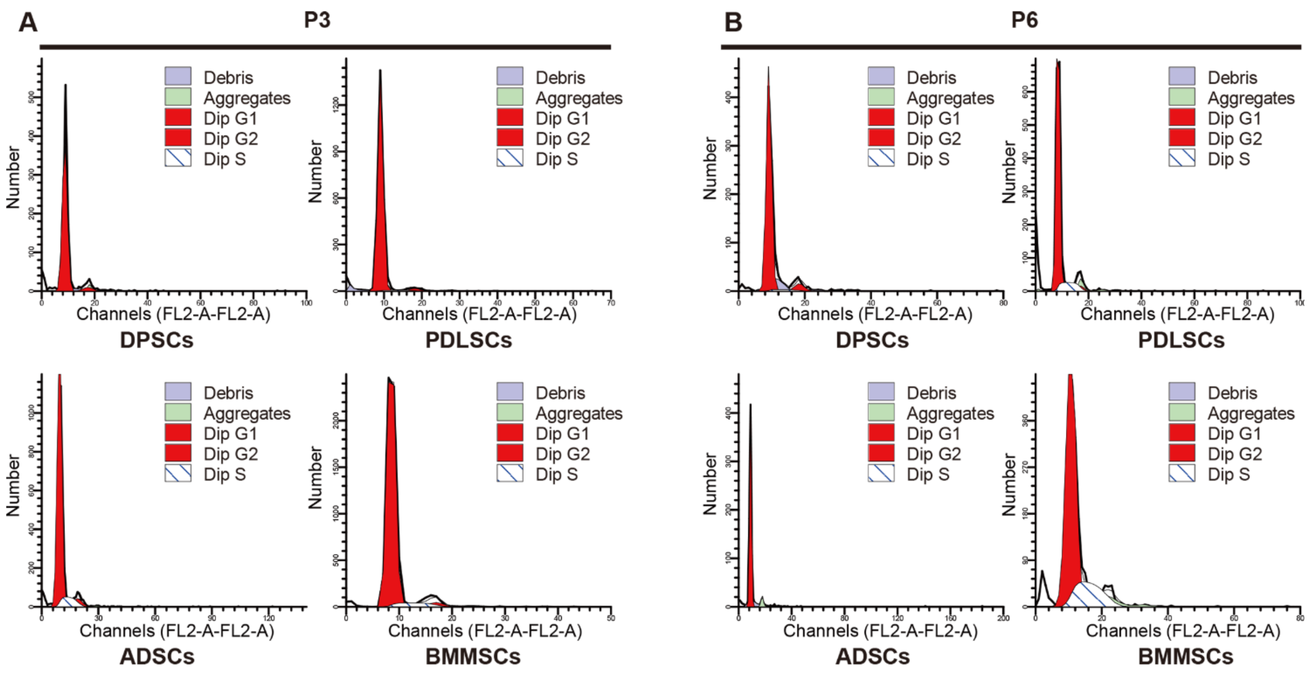

Supplementary Figure 1. Cell cycle analysis of DPSCs, PDLSCs, ADSCs and BMMSCs. (**A**) Cell cycle analysis of DPSCs, PDLSCs, ADSCs and BMMSCs at passage three. (**B**) Cell cycle analysis of DPSCs, PDLSCs, ADSCs and BMMSCs at passage six.

© 2018. Ma L et al. Published online at http://www.aginganddisease.org/EN/10.14336/AD.2018.0728

SUPPLEMENTARY DATA

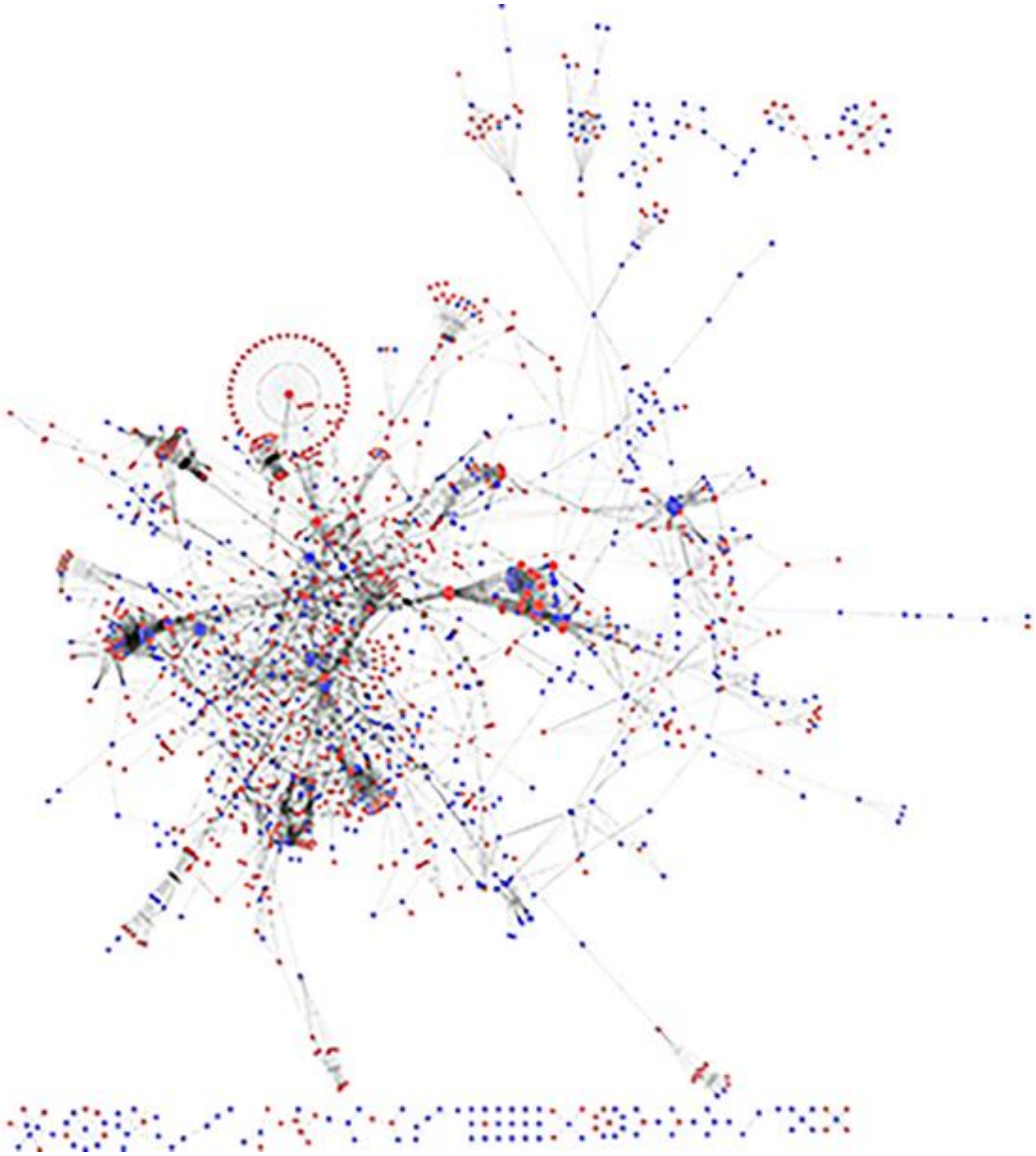

Supplementary Figure 2. Signal-net analysis of the most important candidate genes contributing to the characteristics of DPSCs and BMMSCs. The red represents the upregulated genes and the blue represents the downregulated genes.

© 2018. Ma L et al. Published online at http://www.aginganddisease.org/EN/10.14336/AD.2018.0728

SUPPLEMENTARY DATA

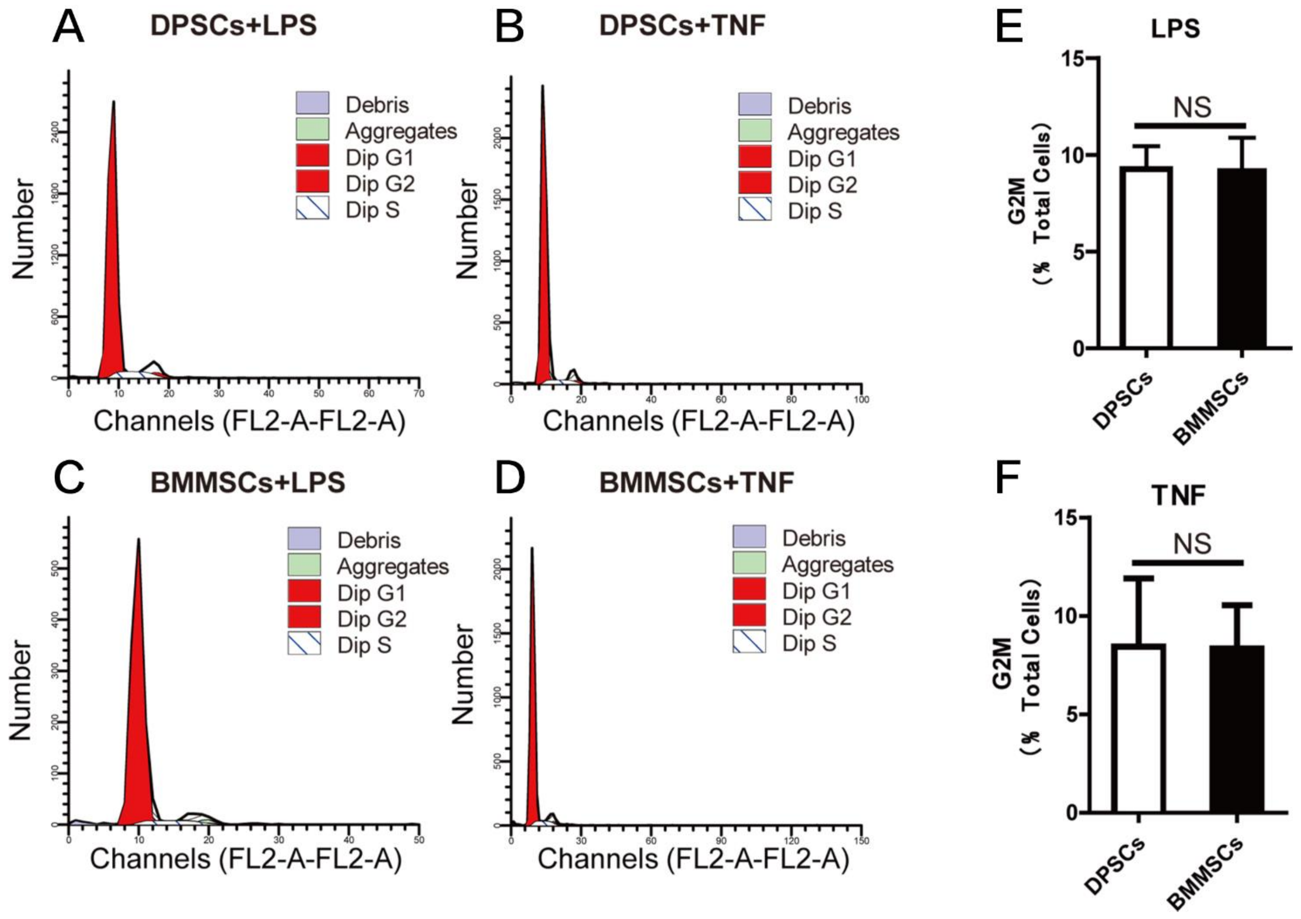

Supplementary Figure 3. Cell cycle analysis of DPSCs and BMMSCs under TNFα and LPS stimulation. **(A)** Cell cycle analysis of DPSCs under LPS stimulation. (**B)** Cell cycle analysis of DPSCs under TNFα stimulation. **(C)** Cell cycle analysis of BMMSCs under LPS stimulation. **(D)** Cell cycle analysis of BMMSCs under TNFα stimulation. **(E, F)** Quantitative analyses of percentage of G2 phase in DPSCs and BMMSCs under TNFα and LPS stimulation. Values are means ± SDs. Student's t-tests were used to determine statistical significance. Error bars represent SDs (n = 10). NS: no significance.

© 2018. Ma L et al. Published online at http://www.aginganddisease.org/EN/10.14336/AD.2018.0728
